# Sky Brightness Evaluation at Concordia Station, Dome C, Antarctica, for Ground-Based Observations of the Solar Corona

**DOI:** 10.1007/s11207-022-01958-x

**Published:** 2022-03-02

**Authors:** Alessandro Liberatore, Gerardo Capobianco, Silvano Fineschi, Giuseppe Massone, Luca Zangrilli, Roberto Susino, Gianalfredo Nicolini

**Affiliations:** 1 OATo – Astrophysical Observatory of Turin, INAF - National Institute for Astrophysics, Via Osservatorio 20, 10025 Pino Torinese, To Italy; 2 Physics Department, University of Torino, Via Pietro Giuria 1, 10125 Torino, Italy

**Keywords:** Sky brightness, Antarctica, Sun, Corona, Coronagraphy

## Abstract

The evaluation of sky characteristics plays a fundamental role for many astrophysical experiments and ground-based observations. In solar physics, the main requirement for such observations is a very low sky brightness value, less than $10^{-6}$ of the solar disk brightness ($\mathrm{B}_{\odot }$). Few places match such a requirement for ground-based, out-of-eclipse coronagraphic measurements. One of these places is, for instance, the Mauna Loa Observatory ($\approx 3400~\mbox{m}$ a.s.l.). Another candidate coronagraphic site is the Dome C plateau in Antarctica. In this article, we show the first results of the sky brightness measurements at Dome C with the Extreme Solar Coronagraphy Antarctic Program Experiment (ESCAPE) at the Italian–French Concordia Station, on Dome C, Antarctica ($\approx 3300~\mbox{m}$ a.s.l.) during the 34th and 35th summer Campaigns of the Italian Piano Nazionale Ricerche Antartiche (PNRA). The sky brightness measurements were carried out with the internally occulted Antarctic coronagraph AntarctiCor. In optimal atmospheric conditions the sky brightness of Dome C has reached values of the order of 1.0 – $0.7 \times 10^{-6}~\mathrm{B}_{\odot }$.

## Introduction

The Sun has an atmosphere divided into several layers. The outermost is called the solar corona. It consists of plasma at very high temperatures (up to $10^{6}$ K), which extends millions of kilometers into outer space. The solar corona results from three different main contributions: the K-Corona, due to Thomson scattering by the free coronal electrons of the photospheric radiation; the F-Corona, due to diffusion of solar radiation by dust particles; and the E-Corona due to emission processes by coronal ions identified as “forbidden” lines.

**Figure 1:**
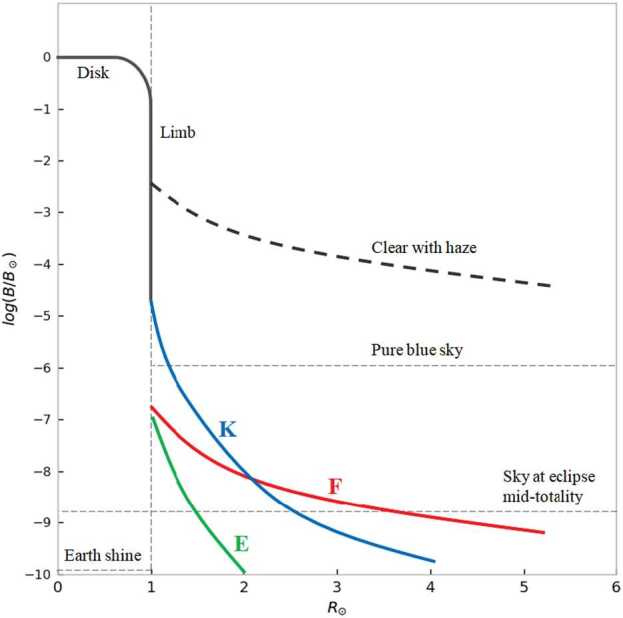
Brightness of the different solar corona components (${B}/\mathrm{B}_{\odot }$) as a function of the heliocentric distance. At least a $B_{\mathrm{sky}} \approx 10^{6} {B}_{\odot}$ is necessary to have a coronagraphic sky.

The brightness of each component decreases with a power law moving away from the Sun (Phillips, 1992; November and Koutchmy, 1996). Figure 1 shows that the brightness of the sky (“clear with haze”) makes ground-based observations of the solar corona difficult. Total solar eclipses give the opportunity to observe the corona with a reduced sky brightness. However, the short duration of these events (max 7.5 minutes), the possibility of adverse weather conditions, and the frequent need of accessing remote locations for the observing sites make it difficult to carry out continuous and detailed coronal studies. A “pure blue sky” for ground-based coronagraphic observations is defined as $\approx 10^{-6}$ of the Sun’s disk brightness ($\mathrm{B}/\mathrm{B}_{\odot }$). Ground-based observations of the solar corona were made possible with the development of the internally occulted coronagraph by Lyot (1932).

Mauna Loa in the Big Island of Hawaii is a coronagraphic site hosting the Mauna Loa Solar Observatory (MLSO) operated by the US High Altitude Observatory. MLSO can carry out systematic observations of the solar corona thanks to a sky-brightness value of $\approx 1-5\times 10^{-6}~\mbox{B}_{\odot }$ for different wavelengths (Tomczyk and Elmore, 2015). One of the goals of the **E**xtreme **S**olar **C**oronagraphy **A**ntarctic **P**rogram **E**xperiment (ESCAPE: Fineschi et al., 2019a) is the determination in Antarctica of a location with a “coronagraphic sky” that would allow systematic ground-based observations of the solar corona. In the next section, we introduce the ESCAPE project instrumentation and goals (Section 2) and show the results of sky-brightness measurements (Section 3) during the 34th (austral summer 2018/2019) and 35th (austral summer 2019/2020) Italian Campaign in Antarctica, Concordia Station, Dome C plateau, at $\approx 3 233~\mbox{m}$ above sea level.

## ESCAPE Project

**Figure 2:**
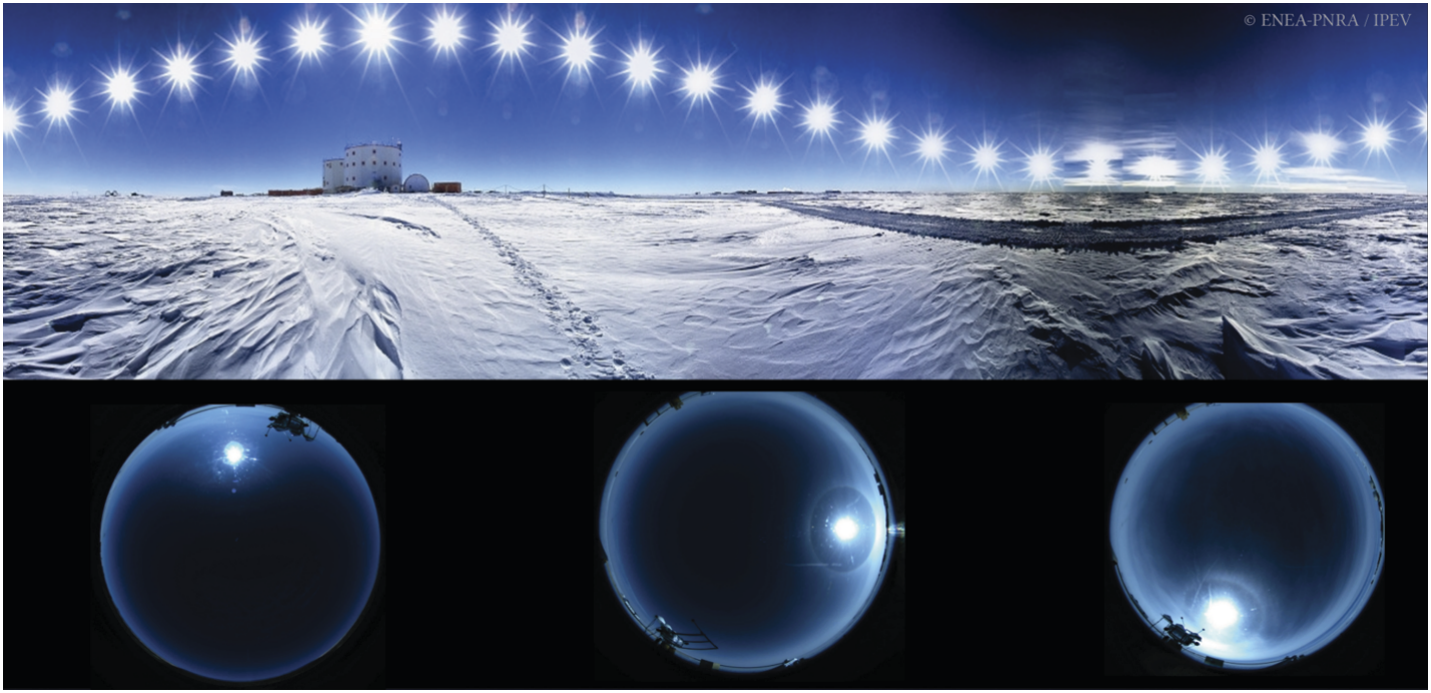
Composition of hourly images showing the Sun position at Concordia Base during the Antarctic summer (Credits: Guillaume Dargaud). In the bottom, 360° images from Baseline Surface Radiation Network (BSRN) project PI, Dr. A. Lupi (Lupi, 2021). It is possible to observe a very good sky condition on the left image. In the middle the presence of a solar halo is a sign of ice crystals in the atmosphere, which can potentially compromise observations by producing straylight. Worst sky conditions are shown on the right with a slight cloud cover as well. By using the full sky camera it is possible to have a general view of sky conditions to avoid the worst days.

Antarctica offers a great opportunity for ground-based observations of the solar corona. Actually, the high altitude of the Antarctic plateau of Dome C ($\approx 3 233~\mbox{m}$ a.s.l.), the high latitude (75° 06′ S, 123° 20′ E), the large amount of daily hours of observations during the Antarctic summer (Figure 2), and the almost total absence of anthropic pollution are necessary conditions for a low sky brightness.

First attempts to characterize the sky brightness at Dome C were performed in 2008 by the pioneering observations of J. Arnaud (Faurobert, Arnaud, and Vernisse, 2012). One of the goals of the ESCAPE Project, at the Italian–French Station Concordia, is to quantitatively evaluate the sky brightness at the Dome C plateau. Within the ESCAPE project, we developed an internally occulted antarctic coronagraph (AntarctiCor) for observations of the K-corona polarized brightness ($pB$) generated by Thomson scattering of photospheric light of coronal free electrons for the determination of the coronal electron density (Van De Hulst, 1950).

**Figure 3:**
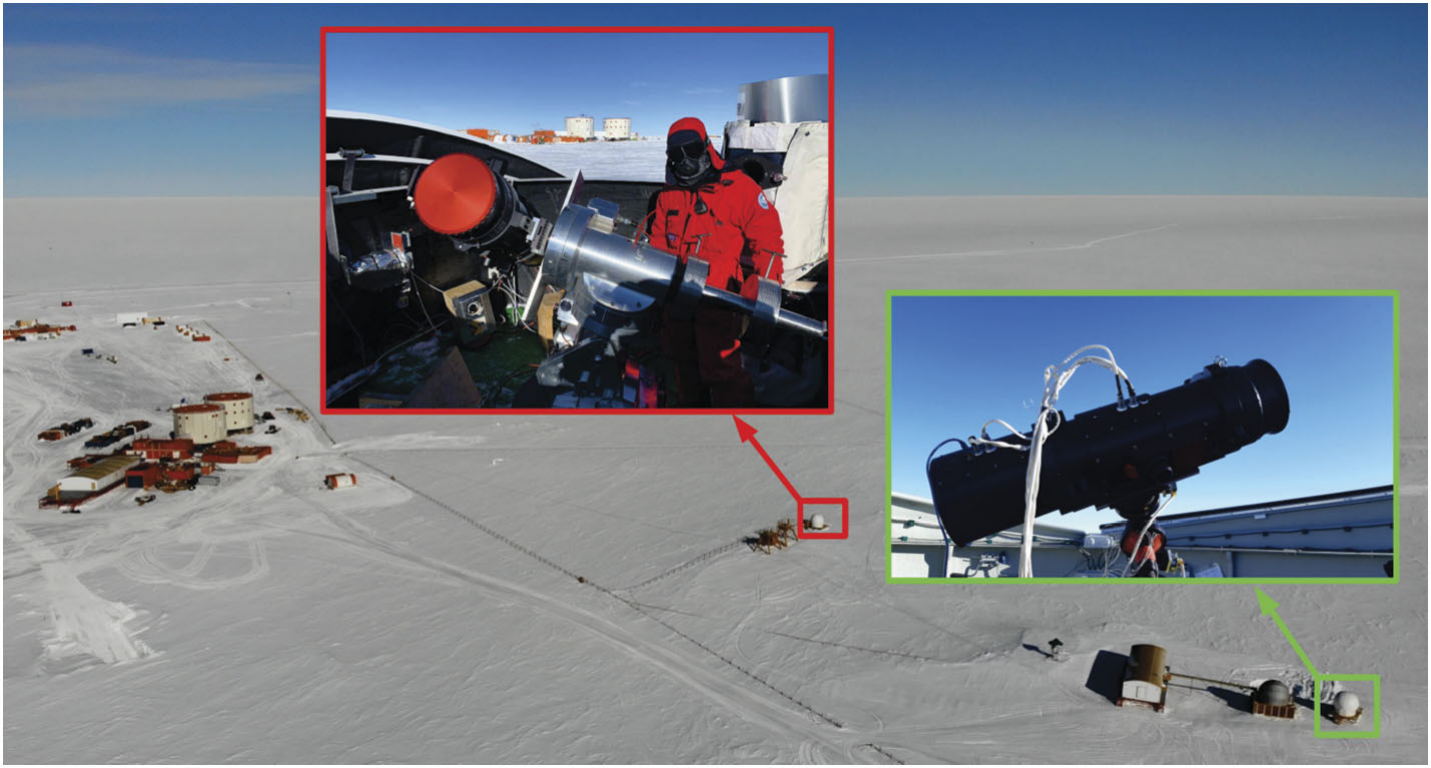
AntarctiCor hosted by the Antarctic Search for Transiting ExoPlanet (ASTEP) project equatorial mount during the 34th Campaign (red box) and in the Baeder dome during the 35th Campaign (green box) at Concordia station for the ESCAPE project (Credits: A. Liberatore and G. Capobianco @PNRA/IPEV).

In the following section, we report the sky-brightness measurements. The coronal images are still under analysis. More details about ESCAPE and its science objectives can be found in Fineschi et al. (2019a). The evaluation of the sky brightness was performed during the 34th and 35th Italian Campaigns in Antarctica by using the Antarctica solar Coronagraph AntarctiCor (Figure 3).

### Antarctic Coronagraph Instrument (AntarctiCor)

**Figure 4:**
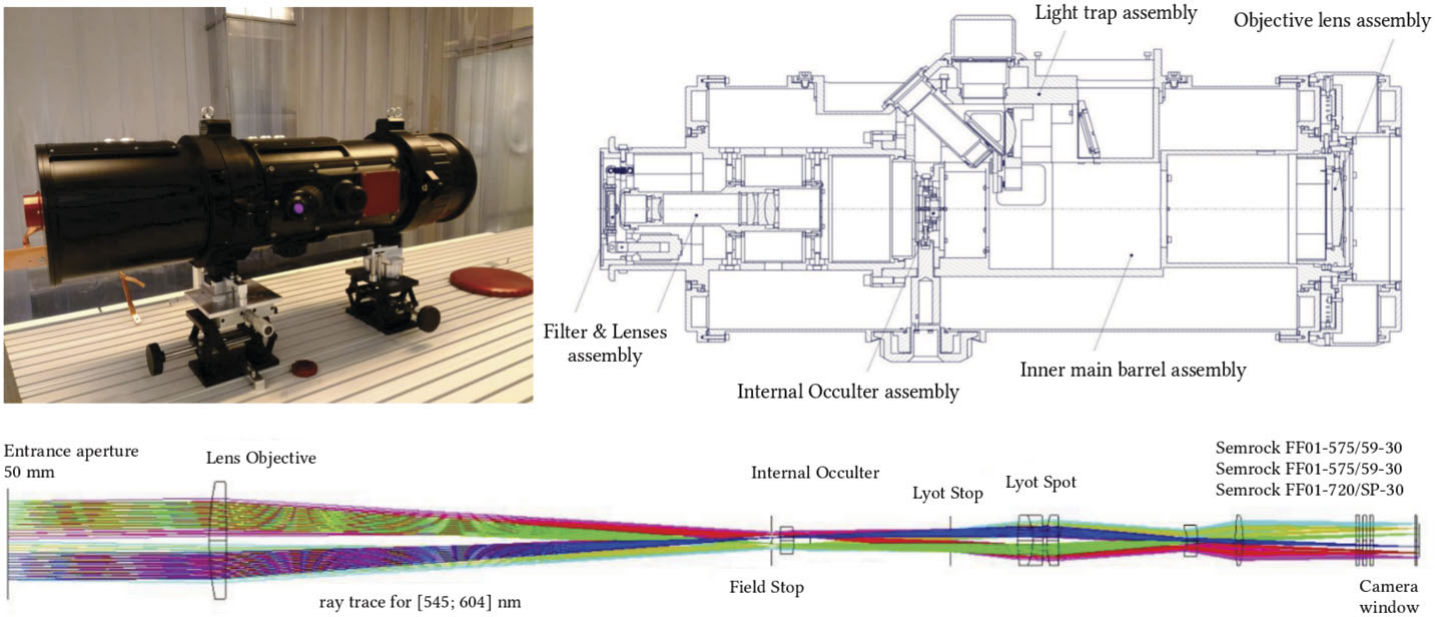
Top left: AntarctiCor in the INAF Optical Payload Systems facility (OPSys) – clean room ISO 5 in Turin (Italy) for tests and calibrations (Fineschi et al. 2019b). The main subassembly diagram (top right panel) comprises: 1. objective lens assembly, 2. inner main barrel assembly, 3. internal occulter assembly, 4. lenses assembly, 5. filter assembly, 6. light trap assembly, 7. microscope assembly. Bottom: AntarctiCor ray tracing for the wide-band $(591 \pm 5)~\mathrm{nm}$ optical path.

The instrument deployed during both campaigns was the Antarctica Solar Coronagraph AntarctiCor (Figure 4). The main features of the instrument are summarized in Table 1. It is a classical Lyot internally occulted coronagraph (Lyot, 1932) based on the externally occulted ASPIICS (Association de Satellites pour l’Imagerie et l’Interferométrie de la Couronne Solaire) coronagraph for the European Space Agency (ESA) formation-flying PROBA-3 (Project for On-Board Autonomy-3) mission (Galy et al., 2015).

**Table 1:** AntarctiCor characteristics (Fineschi et al. 2019b).

Telescope design	Classical internally occulted Lyot coronagraph (Lyot, 1932)
Aperture	50 mm
Eff. Focal Length	700 mm
f/ratio	14
Spectral Ranges	$(591 \pm 5)~\mathrm{nm}$, see Figure 5
Camera type	Interline transfer CCD PolarCam; model: U4 (Zecchino, 2017)
Camera format	1950 × 1950 pixels
Pixel size	$7.4~\upmu \mbox{m} \times 7.4~\upmu \mbox{m}$
Plate scale	4.3 arcsec/pixel
Field of View (FoV)	±0.6°$\equiv \pm 2.24~\mathrm{R}_{\odot }$
Polarization analysis	Spatial modulation by linear micropolarizers on the sensor

**Figure 5:**
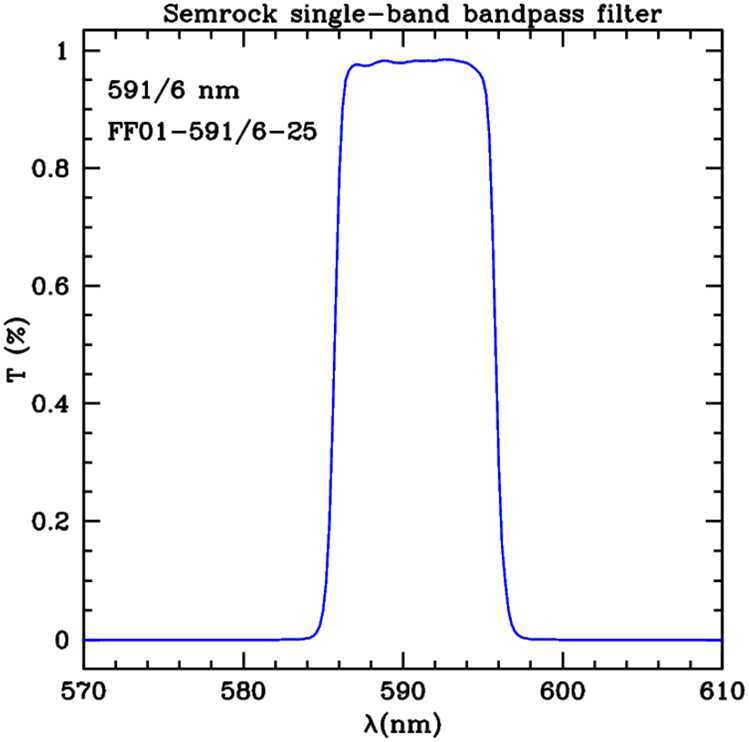
Semrock bandpass filter transmissivity $(591\pm 5)~\mbox{nm}$.

The main characteristics of the AntarctiCor bandpass filter used during the missions are shown in Figure 5 and Table 2. More information can be found in Semrock (2019).

**Table 2:** Main optical and physical filter specifications (Semrock, 2019).

Specification	Value
Transmission band	$T_{\mathrm{avg}} > 93\%$ at 588 – 594.5$~\mbox{nm}$
Center wavelength	$591.25~\mbox{nm}$
FWHM bandwidth (nominal)	$10~\mbox{nm}$
Transverse dimensions (diameter)	$25~\mbox{mm}$
Filter thickness	$5.0~\mathrm{mm}$

Indeed, the telescope design is derived from that of the ASPIICS space coronagraph (Galy et al., 2015). Some modifications from the original design have been adopted due to the main difference between ASPIICS and AntarctiCor: the former is externally occulted and the latter is internally occulted. For example, since the objective doublet lens of ASPIICS operates in the shadow of the external occulter whereas the AntarctiCor objective is directly illuminated by the Sun-disk light, to minimize the internal reflection in the objective lens, this has been changed into a highly polished singlet, i.e. $0.5~\mbox{nm}$ rms (Fineschi et al. 2019b).

**Figure 6:**
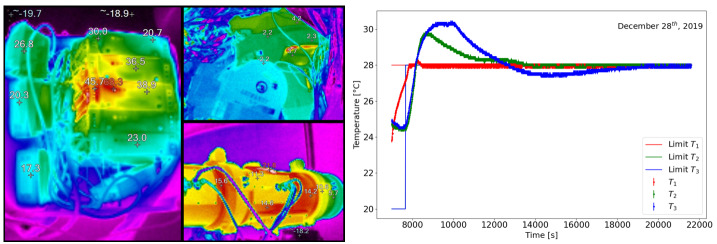
Left: Infrared camera thermal image of the instrumentation. Right: Example of temperature control with a limit set of 28°.

The telescope temperature is acquired in three different points by three PT100 and is controlled by using three heaters with powers of 90 $\mathrm{W}$, 100 $\mathrm{W}$, and 40 $\mathrm{W}$ in the front, central, and rear subassemblies, respectively. The closed-loop heater system keeps the instrument at the set temperature (Figure 6, right). The entire structure is kept at a constant temperature of $\approx 28$°. An infrared camera is used to verify the temperature of the telescope, mount, and the whole instrumentation (Figure 6, left).

### PolarCam Micropolarizer Camera

**Figure 7:**
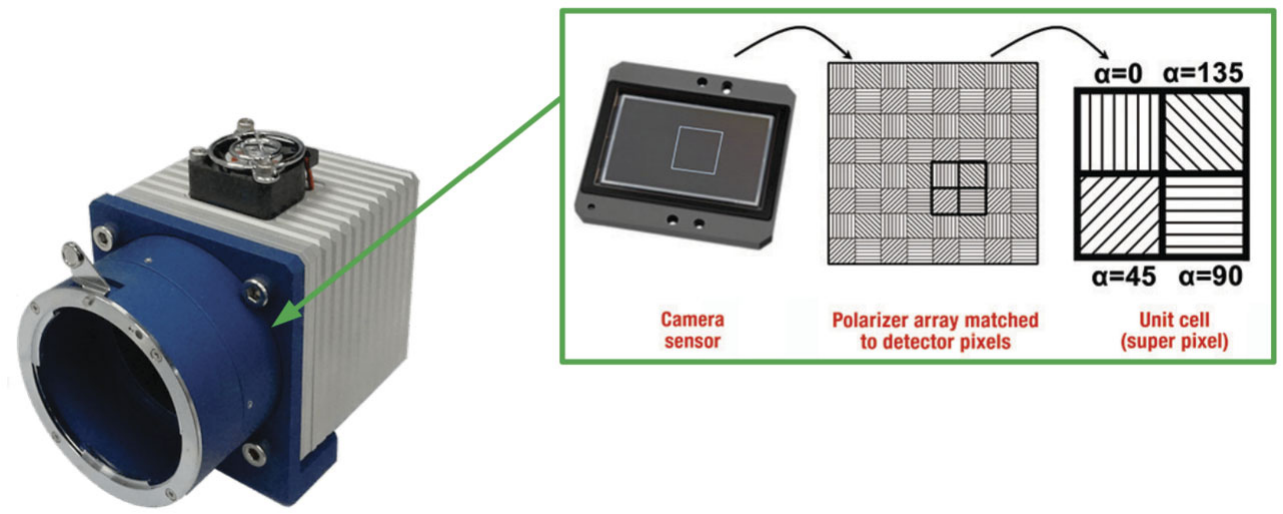
PolarCam detector. An array of linear micropolarizers with different orientation are matching the pixels of the sensor (Zecchino, 2017).

**Figure 8:**
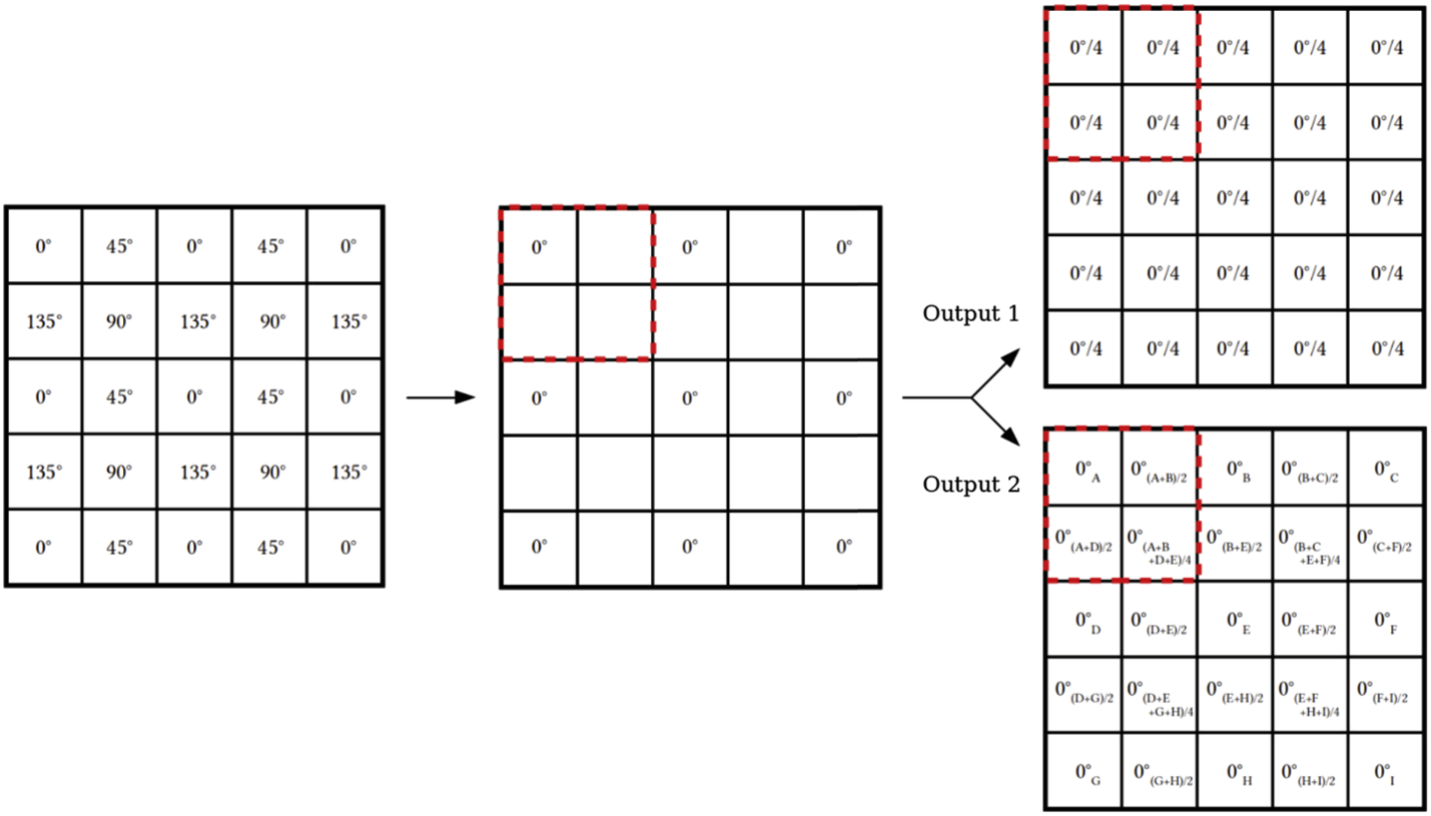
Demosaic example to obtain a polarized image from the original raw image. In this example, the image with a polarization angle of 0° ($I_{0}$) is obtained considering the entire superpixel equal to the pixel value with the micropolarizer at 0° (Output 1) or considering each superpixel obtained averaging the pixel values with the adjacent ones at 0°-pixels (Output 2).

The telescope detector is the PolarCam micropolarizer camera, U4 model.^1^ This camera captures simultaneously four images at multiple polarized angles (0°, 45°, 90°, and 135°) thanks to an array of linear micropolarizers directly applied on the camera sensor. Different micropolarizer orientations match different pixels of the sensor as shown in Figure 7. In this way a single acquisition can return the linear polarization of the image as derived from the Stokes vector parameters: $\textbf{S}= (I,~Q,~U) = (I_{0} + I_{90}, I_{0} - I_{90}, I_{45} - I_{135})$, where $I_{0}$, $I_{90}$, $I_{45}$, $I_{135}$ are the intensities of the linear polarization components at 0°, 90°, 45°, 135°. Indeed, the linearly polarized light requires the measurement of the I, Q, U quantities to be fully characterized (Collett, 1992). For example, it is possible to obtain the polarimetric brightness defined as $pB = \sqrt{Q^{2} + U^{2}}$ or the sky brightness considering the first Stokes parameter. To obtain the single $I_{i}$ (where $i = 0$°, 45°, 90°, 135°), we need to perform a demosaicization process. Once a certain $I_{i}$ has been chosen (e.g. $I_{0}$), we considered the three remaining pixels of each superpixel.^2^ The values of these single pixels are obtained as the averages between the pixels (with the considered polarization $I_{0}$) in each adjacent superpixel as shown in Figure 8, Output 2. The same procedure can be applied to get the images with the other orientations. More information about this camera and its usage can be found in Liberatore et al. (2021) and Zecchino (2017).

## Sky-Brightness Results

During the Antarctic campaigns, systematic images of the sky were acquired to evaluate its brightness. The sky brightness in units of solar disk brightness ($B_{\mathrm{sky}}~[\mbox{B}_{\odot }]$) was measured for the first time during the 34th Campaign (austral summer 2018/2019) and during the full data acquisition during the 35th Campaign (austral summer 2019/2020) at almost regular intervals during all days.^3^ The $B_{\mathrm{sky}}~[{B}_{\odot }]$ can be evaluated by considering the first Stokes parameter $I$ and performing a ratio between what is obtained pointing to the sky ($I_{\mathrm{sky}}$) and what is obtained pointing to the Sun using a diffuser ($I_{\mathrm{diff}}$). Both quantities must be normalized by the respective exposure time $t_{\mathrm{exp}}^{i}$. Then, by considering the diffuser transmissivity $T_{\mathrm{diff}} \approx 28\%$ (EdmundOptics, 2019) we obtain

1 $$ \frac{\overline{I}_{\mathrm{sky}}}{\overline{I}_{\mathrm{diff}}}= \frac{I_{\mathrm{sky}}/t_{\mathrm{exp}}^{\mathrm{sky}}}{I_{\mathrm{diff}}/t_{\mathrm{exp}}^{\mathrm{diff}}} = \frac{B_{\mathrm{sky}}}{(B_{\odot }\cdot T_{\mathrm{diff}})}. $$

Finally, by considering the light scattered over the solid angle by the diffuser over the Sun angular radius ($\vartheta = 16'$) we obtain

2 $$ \frac{\Omega _{\odot }}{2\pi } = \int ^{2\pi }_{0}\int ^{\vartheta }_{0} \frac{\sin {\vartheta '}}{2\pi }d\vartheta 'd\varphi = 1-\cos {\vartheta } = 1.083\times 10^{-5}, $$

and the resulting sky brightness $B_{\mathrm{sky}}~[\mathrm{B}_{\odot }]$ is (Streete, 1989)

3 $$ B_{\mathrm{sky}}[B_{\odot }] = \frac{(I_{\mathrm{sky}/t_{\mathrm{exp}}^{\mathrm{sky}})}}{(I_{\mathrm{diff}}/t_{\mathrm{exp}}^{\mathrm{diff}})} \cdot T_{\mathrm{diff}}\cdot \frac{\Omega _{\odot }}{2\pi }. $$

**Figure 9:**
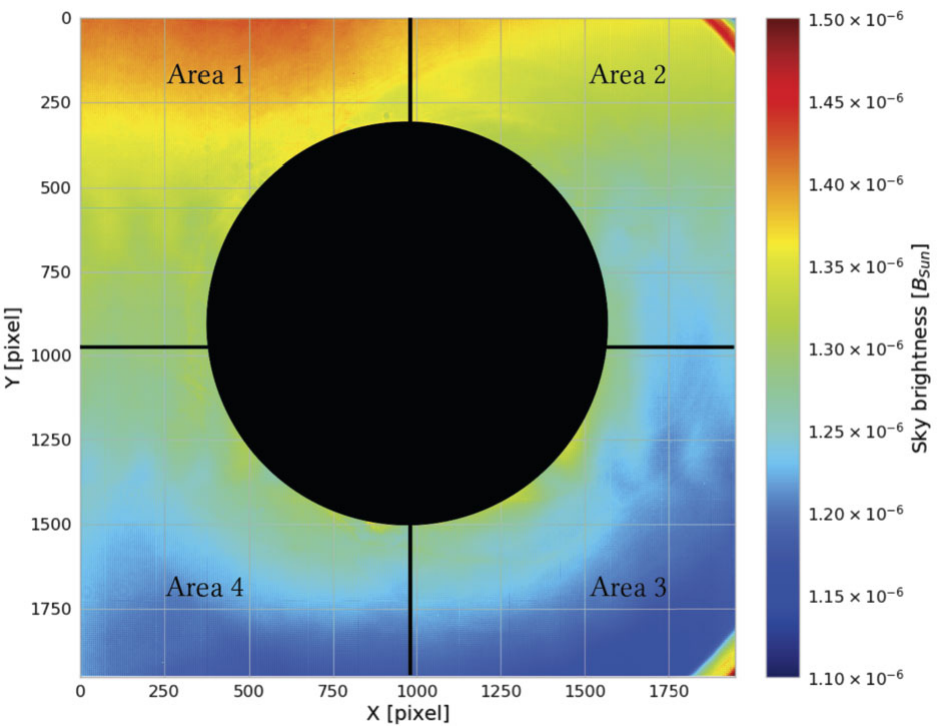
Example of the measured sky brightness (Dome C, Concordia Station, Antarctica) from the 34th Campaign. The entire frame is divided into 4 different pads. The final sky brightness is obtained by averaging the 4 areas.

In particular, in our case the acquired $B_{\mathrm{sky}}~[B_{\odot }]$ frame was divided into four different regions (the areas in Figure 9) and the final brightness was obtained by averaging them:

4 $$ B_{\mathrm{sky}}[B_{\odot }] = \frac{\sum _{i} B_{\mathrm{sky}}^{i}}{4} \quad (i = 1, 2, 3, 4). $$

For each $B_{\mathrm{sky}}$, we evaluate the dispersion values as the standard deviation over the pixels in a considered area (e.g. $\sigma $ over Area 1) quadratically added (and then divided by a factor of 4) to the standard deviations obtained in the other three regions (Area 2, 3, 4).

A so-called “pure blue sky” (i.e. $B_{\mathrm{sky}} \approx 10^{6} B_{\odot}$) is necessary to carry out ground-based observations of the corona (Fracastoro, 1948; Fracastoro and Righini, 1949; Elmore, 2007).

Figure 1 shows the sky brightness in $\mathrm{B}_{\odot }$ units, measurements obtained during the 35th Italian Antarctic Campaign (2019 – 2020) at Concordia Station, Dome C.

From the images acquired during the 34th Campaign on January 08, 2019 (pointing at RA: 22° 42’ 32.3”, Dec: −22° 11’ 22”) and January 09, 2019 (pointing at RA: 22° 38’ 33.3”, Dec: −22° 18’ 19”), we obtain that

5 $$ B_{\mathrm{sky}}= (1.2 \pm 0.1)\times 10^{-6}\ \mbox{B}_{\odot }. $$

**Figure 10:**
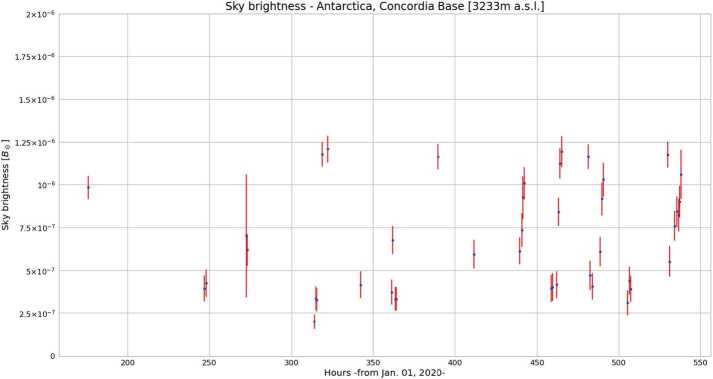
Sky-brightness$~[\mathrm{B}_{\odot }]$ measurements obtained during the 35th Italian Antarctic Campaign (2019 – 2020), Concordia Station, Dome C, $\approx 3230~\mbox{m}$ a.s.l., Antarctica. On the $x$-axis the acquisition UTC time from January 1, 2020, to January 22, 2020. The bars represent the dispersion values obtained by considering the standard deviations for each of the four different detector frame areas.

During the 35th Campaign, it was possible to perform more systematic sky-brightness measurements. Their summary is shown in Figure 10. Averaging over the different values, we obtain

6 $$ \bar{B}_{\mathrm{sky}} = (6.9 \pm 0.2) \times 10^{-7}\ \mathrm{B}_{\odot }. $$

During these measurements, Dome C showed the characteristics of a “coronagraphic sky”. Sometimes, the presence of clouds, high wind, or excessive suspended ice in the atmosphere made it impossible to perform good observations. On the other hand, during this campaign, we evaluated a percentage of good weather days around the 80%!

**Figure 11:**
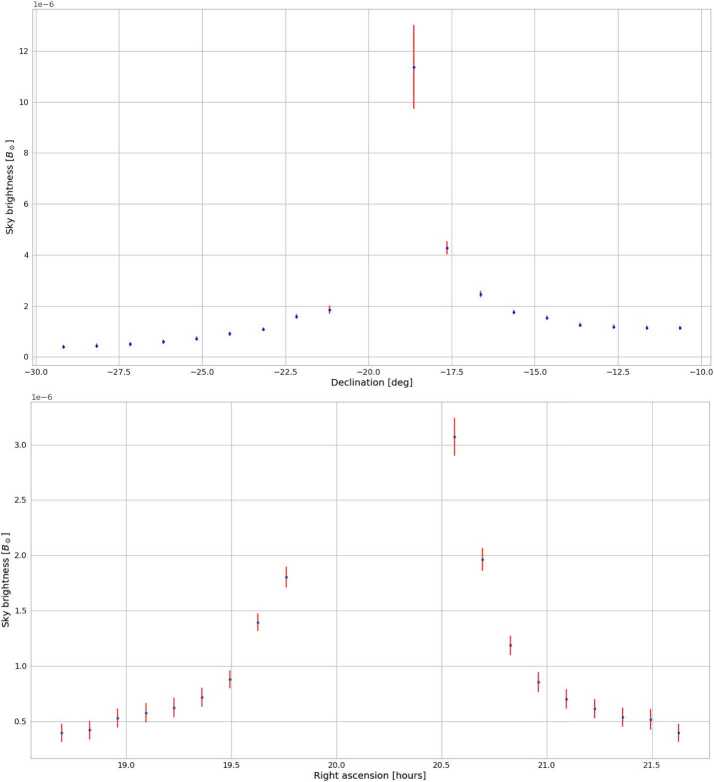
Sky-brightness$~[\mathrm{B}_{\odot }]$ measurements obtained during the 35th Italian Antarctic Campaign (2019 – 2020), Concordia Station, Dome C, $\approx 3230~\mbox{m}$ a.s.l., Antarctica, for different declination values (top) and different right ascension ones (bottom) on January 1, 2020. The Sun position (J200 system) was AR: 20° 09′ 38′′, Dec: −20° 05′ 57′′.

During the same mission, we evaluated the sky brightness for different right ascensions (RA) and declinations (DEC) for a fixed day (see Figure 11). The values of measured $B_{\mathrm{sky}}$ are consistent with those obtained in Figure 10 and, as expected, it is possible to see a decrease of sky brightness moving away from the Sun. The closest measurements to the solar limb are at about 0.7°; e.g., for the declination, the closest measurement was at 1° from Sun center (Figure 11 top), so $1 - 0.26 = 0.74$° from the solar limb (where 0.26° is the solar radii). We cannot really go much closer than 1° from Sun center due to the instrument filed of view (FOV) along the $x$- and $y$-axes. The instrument FOV is $\pm 0.6$° (Table 1) and we need that the Sun is completely out to take sky-brightness measurements.

These results show that the Antarctic sky at Dome C, where Concordia Station is located, has periods when it can be considered to have a “coronagraphic sky” (e.g. Fracastoro 1948; Fracastoro and Righini 1949; Elmore 2007; and Figure 1).

## Conclusion

In the present work, we presented the first results from the ESCAPE project. In particular, after a description of the project and its main goal, we described the instrumentation used during the Antarctic missions and its characteristics. We described the internally occulted coronagraph AntarctiCor and its innovative detector with arrays of micropolarizers for linear polarization imaging. Then we described how we evaluated the sky brightness $\bar{B}_{\mathrm{sky}}$ at the Concordia Station (Dome C plateau, Antarctica, coord. 75° 06′ S; 123° 20′ E) at an altitude of $\approx 3300~\mbox{m}$ above sea level. The $\bar{B}_{\mathrm{sky}}$ value was obtained during two different missions. In the first one (34th Italian Campaign to Antarctica, austral summer 2018/19), a sky brightness of $\approx 1.0 \times 10^{-6}\ \mathrm{B}_{\odot }$ was measured. Due to a logistic problem, just few acquisitions during the mission were possible (from 18 January 2019 to 19 January 2019). These sky-brightness values have been obtained for a fixed distance from the Sun. Subsequently, during the 35th mission, we performed more systematic measurements (from 1 January 2020 to 22 January 2020). In addition, we performed measurements of the sky brightness not only for a fixed distance from the Sun, but for different declination and different right ascension values. In this second campaign, we obtained that $\bar{B}_{\mathrm{sky}} \approx 7.0 \times 10^{-7}\ \mathrm{B}_{\odot }$. Both results quantitatively demonstrate, for the first time, the quality of the Dome C site for coronagraphic observations. We can conclude that the Antarctic sky at Concordia Station shows the characteristics of a “coronagraphic sky” (i.e. $B_{\mathrm{sky}} < 10^{6}~\mbox{B}_{\odot }$). This holds the promises for Concordia Station to host a permanent coronagraph observatory for continuous studies of the solar corona during the Austral summers.

## Data Availability

The datasets generated during and/or analyzed during the current study are available from the corresponding author on reasonable request.

## Footnotes

1 The PolarCam^©^ is manufactured by 4-D Technology Corporation, Arizona, United States (4D-Technology, 2021).

2 A superpixel is the “pixel” obtained by considering the four adjacent pixels with different micropolarizer orientations (0°, 45°, 90°, and 135°).

3 The 36th Campaign (austral summer 2020/2021) was limited to logistical activities due to the COVID-19 pandemic.
